# Editorial: Biorisk management, laboratory acquired infections and clinical containment

**DOI:** 10.3389/fpubh.2023.1127856

**Published:** 2023-01-17

**Authors:** Kashif Ali, Furqan Kabir, Esmeralda Meyer

**Affiliations:** ^1^Department of Biosciences, Faculty of Life Sciences, Shaheed Zulfiqar Ali Bhutto Institute of Science and Technology, Karachi, Pakistan; ^2^Infectious Diseases Research Laboratory (IDRL), Department of Paediatrics and Child Health, The Aga Khan University, Karachi, Pakistan; ^3^Institutional Animal Care and Use Committee (IACUC), Research Compliance & Regulatory Affairs, Emory University, Atlanta, CO, United States

**Keywords:** biosafety and biosecurity, diagnostic laboratories, policy and guidelines, communication strategy, handwashing

The field of Biosafety is as old as Microbiology but gained significant attention when Arnold G. Wedum published articles on applied biosafety and risk assessments. Biorisk Management is a framework that encompasses both biosafety and biosecurity and enables an organization for the identification, assessment, mitigation, evaluation, and communication of the inherent biosafety and biosecurity risks. Biorisk management is gaining importance during the recent pandemic with the promise of mitigating laboratory acquired infections through multi-factoral approaches.

This Research Topic aims to cover promising, recent, and novel research trends in the domains of Biorisk Management, Laboratory Acquired Infections, Biosafety and Biosecurity. Specifically, it presents comprehensive reviews on new frontiers in biosafety and biosecurity, different approaches for the biorisk assessment, and biorisk management of genetic editing or microorganisms. The research articles included in the topic are mainly covering the areas of communication strategies, biocontainment in poultry industry, assessment and ways to improve the current biosafety and biosecurity situation in diagnostic and research laboratory, along with the importance to relevant training on these protocols.

In an interesting review article, Raybould present an overview on three new frontiers in biosafety and biosecurity and how biotechnology can be helpful in this regard. The author emphasized on the continuous improvement in policy and decision making to maximize the balance between opportunity and risk in applying biotechnology to solve societal challenges. He presented political leadership, innovative legislation, and responsible business and civil society participation as the new areas which should now be focused to achieve the overall objective of biosafety and biosecurity.

Bellati et al. advocate for the use integrated approach against the traditional approach of biosafety for the effective risk assessment in a laboratory, as the integrated approach contain multiple psychological and organizational factors. These factors should not be considered as secondary but recognized as fundamental for risk assessment.

Gene editing platforms have changed genetics in general and public health in particular. Despite its obvious benefits, it's widely debated for its hazards and uncertainty. Kalidasan and Theva Das highlight the problems raised by modern biotechnology in Malaysia concerning gene editing legislation, biosafety, and biosecurity. Although, in Malaysia, stem cell and cell-based therapies have standards and guidelines, appropriate legal framework for gene editing is still the need. In the same context, biosafety regulations are created to promote biotechnology while minimizing environmental and health dangers. It is also important to address the potential use of GMOs as bioweapons. Multiple international frameworks can be helpful for Malaysia to successfully implement gene editing by developing thorough guidelines, legal policies, and standards.

Merrill et al. highlight the impact of communication strategy with the biosecurity. As the COVID-19 pandemic continues worldwide, it's become evident that good communication strategies regarding disease transmission risks and protective practices is vital but not universally understood. Illnesses resulted in animal fatalities cost hundreds of millions of dollars annually to the US hog industry. Biosecurity methods can lower these expenditures. Effective Biosecurity depends on constant execution and effected by human decision-making. Using an experimental game, Merrill et al. quantify how different messages of disease incursions affect the compliance of biosecurity procedures. The study shows that graphical communications mixed with linguistic terms denoting infection risk levels are more successful for guaranteeing biosecurity compliance than simple linguistic phrases or graphical messages with numeric risk levels.

Biosecurity techniques are extensively promoted to reduce the economic loss in poultry industry. In this article, Otte et al. employ a home economics viewpoint to examine village poultry keepers' biosecurity investments. The 2012/13 Tanzania National Panel Survey (TZ-NPS) covered 1,228 poultry-keeping households and in most which, disease caused more than half of bird losses. Given that chickens rarely contributed more than 10% of annual household income, 95% of households lost 10% of revenue due to disease. The value of poultry varies by gender, and the total amount may disguise intra-household differences. The “typical” village poultry-keeping household may not prioritize poultry investments, even if cost-effective. When disease risks touch the wider community and generate major externalities, poultry keepers must be supported by wider societal measures.

Campylobacter is the largest cause of bacterial diarrhea in humans, and chicken meat products are considered as a major source. Due to the prevalence of Campylobacter in poultry farms, biosecurity is the key area for intervention. A research study by Royden et al. examine farmers' biosecurity attitudes and found impediments to effective adoption. Staff members, farmers, managers, and workers with varying industry expertise were interviewed. Broiler farmers recognize the relevance of Campylobacter and the farm-to-fork chain's responsibility to reduce Campylobacter contamination of chicken meat for public health. This shows the improved status of participants' biosecurity awareness and the industry-wide focus on Campylobacter control. Participants questioned the efficiency of current biosecurity efforts in reducing Campylobacter. The study revealed that more farmer education is needed about biosecurity initiatives, including Campylobacter management.

Muhammad et al. study the current situation of diagnostic and research laboratories in Pakistan with respect to biosafety and biosecurity. They identified that diagnostic and research laboratories have made considerable gains in biosafety and biosecurity due to increased biorisk management knowledge. A total of 30 laboratories, 11 diagnostic, and 19 research labs, are surveyed and it is identified that research laboratories are better in personal protective equipment, biosafety behavior, waste management, biosafety and biosecurity measures, trainings, and safety and health services than diagnostic laboratories.

Miguita et al. suggest that with adequate control measures of biosafety, including patient telemonitoring, proper use of personal protection equipment, and sanitization of surfaces, cross infection of SARS-CoV-2 can be avoided and dental practice can be safely executed.

Vennis et al. provide a comprehensive overview of the worldwide legal biosecurity framework to biosecurity academics, policymakers, civil servants, and practitioners in order to provide a better understanding of the existing international instruments of biosecurity. The paper offers practical applications for and improves multidisciplinary capacity to prevent, identify, and respond to the spread of infectious disease.

Handwashing in Good Microbiological Practices & Procedures (GMPP) is considered as the most important risk control measure. In a simple but effective study, Sarwar et al. demonstrate that how to avoid the use of paper towel for closing the tap in a resource-limited settings. This paper describes a hand-washing procedure that not only doesn't require paper towels but also report easy execution and elevated handwashing compliance.

To emphasize the importance of relevant biosafety training, Qasmi et al. demonstrated that how an effective international virtual training can improve the awareness and knowledge of the laboratory professionals and students.

The Guest Editors would like to express their gratitude to all the authors and reviewers of this Research Topic and acknowledge their hard work and dedication toward the area of biosafety and biosecurity. The Guest Editors believe that the presented researches will encourage the generation of more knowledge and valuable research in the fields of biorisk management, laboratory acquired infection, and clinical containment.

## Author contributions

This editorial is drafted by KA and FK and reviewed by EM. All authors contributed to the article and approved the submitted version.

